# Toward developmental models of psychiatric disorders in zebrafish

**DOI:** 10.3389/fncir.2013.00079

**Published:** 2013-04-26

**Authors:** William H. J. Norton

**Affiliations:** Department of Biology, College of Medicine, Biological Sciences and Psychiatry, University of LeicesterLeicester, UK

**Keywords:** zebrafish, psychiatric disorders, development, attention-deficit/hyperactivity disorder, autism spectrum disorder, schizophrenia, mental retardation

## Abstract

Psychiatric disorders are a diverse set of diseases that affect all aspects of mental function including social interaction, thinking, feeling, and mood. Although psychiatric disorders place a large economic burden on society, the drugs available to treat them are often palliative with variable efficacy and intolerable side-effects. The development of novel drugs has been hindered by a lack of knowledge about the etiology of these diseases. It is thus necessary to further investigate psychiatric disorders using a combination of human molecular genetics, gene-by-environment studies, *in vitro* pharmacological and biochemistry experiments, animal models, and investigation of the non-biological basis of these diseases, such as environmental effects. Many psychiatric disorders, including autism spectrum disorder, attention-deficit/hyperactivity disorder, mental retardation, and schizophrenia can be triggered by alterations to neural development. The zebrafish is a popular model for developmental biology that is increasingly used to study human disease. Recent work has extended this approach to examine psychiatric disorders as well. However, since psychiatric disorders affect complex mental functions that might be human specific, it is not possible to fully model them in fish. In this review, I will propose that the suitability of zebrafish for developmental studies, and the genetic tools available to manipulate them, provide a powerful model to study the roles of genes that are linked to psychiatric disorders during neural development. The relative speed and ease of conducting experiments in zebrafish can be used to address two areas of future research: the contribution of environmental factors to disease onset, and screening for novel therapeutic compounds.

## INTRODUCTION

Psychiatric disorders are a diverse group of diseases that can affect all aspects of mental function including thinking, feeling, mood, and sociability. Psychiatric disorders place a massive strain on society. They are the leading cause of disability in Europe and North America (Eaton, 2008) and rank second in the burden of diseases in established market economies (World Health Organisation [WHO], 2008). However, the drug therapies available to treat psychiatric diseases are often only palliative and have variable efficacy and side-effects. Many of the compounds used to treat psychiatric disorders were discovered serendipitously more than 50 years ago and have not been significantly improved since (WHO, 2008). Furthermore, the high costs associated with developing new treatments, estimated at roughly $1.8 billion per drug, has prompted several major pharmaceutical companies to reduce or cancel their central nervous system research programs (Paul et al., 2010). One of the factors that have hindered the discovery of novel drugs is a lack of knowledge regarding the genetics and neurobiology of these diseases. Further research into the etiology of psychiatric disorders, driven by a combination of human genetic studies and animal models, and taking into account environmental influences, is needed in order to improve drug treatments and develop early prodomic interventions (before disease symptoms are visible) that could prevent or delay disease onset.

## THE ENVIRONMENTAL AND GENETIC BASIS OF PSYCHIATRIC DISORDERS

Psychiatric disorders are caused by interaction of multiple factors that has been described by Gottesman and Gould (2003) as a “ballet that is choreographed over time between the action of multiple genes, environment and epigenetic factors.” Initially, psychiatric disorders were thought to be triggered by the environment with only a limited influence of genes (Cooper, 2001). However, since the advent of genome-wide association studies (GWAS), there has been an increase in the amount of work that focuses on the genetic basis of psychiatric disorders. In parallel, data from twin family and adoption studies have uncovered heritability estimates for diseases such as schizophrenia (81%), attention-deficit/hyperactivity disorder (ADHD, 80%), and autism spectrum disorder (ASD, 70–80%) suggesting a critical role for genetic lesions in these disorders (Sullivan et al., 2003; Bailey et al., 2005; Rosenberg et al., 2009; Banaschewski et al., 2010). Since it is difficult to study psychosocial interactions in animal models, this review will concentrate on the genetics of psychiatric disorders with a focus on alterations to early neural development.

The genetics of psychiatric disorders are often complicated, with a non-Mendelian inheritance pattern and a continuous variation in phenotype suggesting that they might be caused by the action of multiple genes. This observation led to the common disease-common variant (CDCV) hypothesis: inheritance of one variant alone is not enough to cause a disease, but when combined with others a theoretical threshold will be passed and the disease triggered (Mitchell, 2011). As a result of the CDCV hypothesis, GWAS have been used to identify many genes associated with psychiatric disorders. GWAS are based upon the principle that multiple disease-causing variants (such as single nucleotide polymorphisms, SNPs) will be maintained in linkage-disequilibrium and so can be detected in an unbiased manner (Frazer et al., 2009). However, it is often unclear whether a loss or gain of gene function leads to expression of a psychiatric disorder. Following GWAS, mechanistic studies are then needed to show that identified variants participate in the disease being studied. Despite their promise, the contribution of GWAS to the understanding of psychiatric disorders has not been very impressive (Gottesman and Gould, 2003). Genes identified by GWAS still only account for a small percentage of the heritability of complex traits (Frazer et al., 2009) with poor correspondence of data across studies (Mitchell and Porteus, 2011). This is due to a combination of the modest effect of causative mutations on disease susceptibility, under-powered studies with small sample sizes, and the need to take environmental influences into account when studying genetic lesions.

Recent research has provided evidence that some psychiatric disorders, including schizophrenia, ADHD, autism, and mental retardation can also be triggered by mutations in single genes [sometimes referred to as the common disease rare variant (CDRV) hypothesis; Sebat, 2007; Walsh et al., 2008; Elia et al., 2009; Girirajan and Eichler, 2010; Williams et al., 2010; Geschwind, 2011; Lesch et al., 2011; Lionel et al., 2011; Veltman and Brunner, 2012]. Studies of families that suffer from psychiatric disorders often reveal *de novo* (or private) mutations which are unique to a given population or family. In fact, current estimates suggest that on average up to 74 novel mutations may occur per genome per generation in the non-disease population (Veltman and Brunner, 2012), with psychiatric disease families showing even higher than normal rates (Girard et al., 2011). These mutations are rare (comprising less than 1% of the minor allele frequency) and can either represent SNPs or be contained within copy number variations (CNVs) – deletions or duplications that can affect one or more genes at the same time (Sebat et al., 2009). CNVs occur quite frequently and have a mutation rate that is three to four times higher than other genomic areas, accounting for more genetic variation than other types of polymorphism (Cook and Scherer, 2008). Mutations can alter gene function in several ways, each of which can potentially lead to a psychiatric phenotype: they can activate proteins, create abnormal biochemical functions, or abrogate gene activity (Walsh, 1999; Walsh and Engle, 2010). Furthermore, a single gene can sometimes be mutated in multiple positions, each of which can trigger a different disease; whilst psychiatric disorders may be caused by a large number of different mutations, they might paradoxically only be linked to a small number of genes. For example, mutations in *Disrupted in Schizophrenia 1* (*Disc1*) can lead to schizophrenia, bipolar disorder, major depressive disorder, and autism (Porteous et al., 2011). Similarly, a microdeletion at human chromosome locus 22q11 is associated with a range of diseases including schizophrenia and velocardiofacial syndrome, anxiety, depression, ADHD, obsessive-compulsive disorder, and ASDs (Gothelf et al., 2004). Thus, seemingly diverse diseases may share a common genetic basis, making it possible to identify disease-causing variants in known – rather than novel genes in some cases.

The relative contribution of rare or common mutations to disease susceptibility is not known. Psychiatric disorders could be caused by a combination of several types of genetic lesion rather than a single mutation alone (Veltman and Brunner, 2012). Single mutations might predispose patients to mental illness, while other SNP polymorphisms in the genetic background (or mutations in a second critical gene) could alter disease penetrance (Girirajan and Eichler, 2010; Mitchell and Porteus, 2011). Therefore, a single mutation may be necessary but not sufficient to trigger the disorder. Such a combined model would explain the large heterogeneity of symptoms and the low penetrance that is sometimes observed. The potential interaction between rare and common mutations in the etiology of mental disease is reminiscent of the genetics of cancer. According to Knudson’s two-hit hypothesis, cancer can be triggered by the combination of two or more mutations (Knudson, 1971). A recessive germline mutated allele is inherited from one parent, followed by a novel somatic mutation in the same gene (Guidry and Kent, 1999). The combination of common variants and rare mutations associated with psychiatric disorders raises the possibility that psychiatric disorders may also be caused by loss of heterozygosity or by the inheritance of two “hits” in separate genes (Girirajan and Eichler, 2010; Toro et al., 2010). The considerable number of brain areas and processes affected by psychiatric disorders provide a large pool of mutable genes which could lead to expression of a disease (Xu et al., 2008). As an example of this, CNVs in both *NRXN1* and *CNTNAP2* have been linked to Pitt–Hopkins-like syndrome, a familial disease that includes autistic symptoms (Zweier et al., 2009). Patients with Pitt–Hopkins-like syndrome have been found to have lost one copy of *NRXN1* or *CNTNAP2* coupled to a deleterious point mutation in the second allele of the same gene (Toro et al., 2010).

Interestingly, phylogenetic analysis of disease-causing mutations suggests that our current classification of psychiatric disorders may be based upon culturally acceptable behavioral norms. For example, in the case of the ADHD-linked gene *LATROPHILIN3*, the disease-causing variant is ancestral with protective variants arising over time (Arcos-Burgos et al., 2010; Domene et al., 2011). Thus some of the symptoms of ADHD – including hyperactivity and impulsivity – were either advantageous or at least not selected against during evolution. Similar results have been found for *Calretinin* polymorphisms linked to schizophrenia (Farokhashtiani et al., 2011), with disease-causing variants found in other distantly related vertebrate species.

The interaction of genes with environmental factors (including infection, drugs, malnutrition, psychosocial adversity, or obstetric complications) is likely to play a significant role in the risk of suffering from a psychiatric disorder. Genes may predispose people to suffer from a disease, which is triggered when an adverse environment is encountered. Thus the severity of a disease might be determined by the interaction of a single gene with different environmental factors. These environmental factors may also be under genetic control. For example, the genetic variants that cause maternal ADHD also increase the propensity of mothers to drink or smoke (Castellanos et al., 1996; Laucht et al., 2007; Thapar et al., 2009). Furthermore, the number of germline mutations in sperm increases with age, exacerbating the likelihood of suffering from psychiatric disorders such as autism (Veltman and Brunner, 2012). In some cases, environmental influences may ultimately reflect the interaction of the parent’s and child’s genomes in expression of mental illness (Castellanos et al., 1996; Laucht et al., 2007). The gene-by-environment (G × E) interactions that lead to psychiatric disorders are poorly understood. However, a recent study of the human ADHD-risk gene *LATROPHILIN3* suggests that G × E interactions may not have a linear impact upon disease susceptibility (Choudhry et al., 2012). In impoverished environments (such as maternal stress during pregnancy), mutation of *LPHN3* has little contribution to disease onset with environmental factors playing a major role. However, in adequate or enriched environments, polymorphisms in *LPHN3* have a greater chance to trigger the disorder (Choudhry et al., 2012).

## ALTERATIONS TO EMBRYONIC DEVELOPMENT CAN LEAD TO PSYCHIATRIC DISORDERS

Many of the rare mutations linked to psychiatric disorders are found in genes that are active during embryonic development. Subtle disruptions to the homeostasis of normal development can have far-reaching consequences that lead to permanent alterations in the function of the mature brain (Mitchell, 2011). Psychiatric disorders can thus be conceptualized as deviations in the normal trajectory of embryonic development within acceptable noise levels resulting in non-lethal modifications of behavior (West-Eberhard, 2005). Genetic variation and developmental plasticity (including chance events) are a fundamental property of all living organisms and provide the raw material upon which evolution can act. Development thus constitutes a series of branching pathways in which developmental decisions switch between different potential endpoints (West-Eberhard, 2005). For example, dopaminergic neurons show a surprisingly stochastic wiring pattern in larval zebrafish, even though they are thought to be genetically homogenous (Tay et al., 2011). Extreme alterations to embryonic development are likely to be lethal. However, if a mutation coincides with a chance event, a subtle change to embryonic development that leads to a psychiatric disorder may occur. Alterations to neural development can act at the cellular or circuit level. In some cases mutations may cause specific phenotypes linked to neurological disorders, such as the control of cell division, migration, differentiation, and survival, or changes to neurite outgrowth, axon pathfinding, and dendritic architecture (Thornton and Woods, 2009; Valiente and Marin, 2010). Alternatively, mutations may cause more diffuse and variable alterations to brain function: miswiring of neural circuits, disinhibition of local interneurons, or adjustment of normal brain homeostasis leading to pathophysiology (Lisman et al., 2008), a process which has been called “developmental disconnection” in relation to ASDs (Geschwind and Levitt, 2007). Diffuse changes to brain function seem particularly likely to lead to psychiatric diseases when considering that genes do not directly control behavior, but rather act via the formation, connection, and function of neural circuits.

## TRANSLATIONAL MODELS OF PSYCHIATRIC DISORDERS IN ZEBRAFISH

Although recent studies have uncovered many genes linked to psychiatric disorders, only few of them have been experimentally validated. Therefore, mechanistic studies are required in order to investigate whether a loss- or gain-of-function contributes to disease pathology in each case. The complex genetic basis of psychiatric disorders makes it difficult to fully recreate them in animal models. Thus, the challenge of studying these diseases consists of integrating basic molecular data from animals with information about complex human mental functions at the circuit level (Geschwind, 2008). One way to simplify this problem is to measure endophenotypes, neuropsychological or biological markers that correlate to a disease-gene’s activity (Gottesman and Gould, 2003; Kendler and Neale, 2010). An ideal endophenotype should be controlled by a single gene, be associated with expression of the disease in the population and be both heritable and state independent (meaning that it is expressed even when the illness is not active; Rommelse, 2008). Although in animal models endophenotypes have rarely fulfilled all of these criteria, their use may simplify the translation of information to human patients. Furthermore, the division of psychiatric disorders according to endophenotypes may help refine their diagnosis; diseases could thus be reclassified on the basis of their molecular pathology instead of behavioral or psychological symptoms, providing an explanation for comorbidity with other disorders (Gottesman and Gould, 2003).

Despite the difficulty of modeling psychiatric disorders, animal studies still have the potential to give insights into the etiology of mental illness. The advent of tools to manipulate genes has now allowed the creation of animal models that are firmly based upon the genetic pathways underlying a disease. A perfect animal model should have three main attributes: construct validity (meaning that it conforms to the underlying rationale of the disease), face validity (mimicking some of the characteristics of the disease), and predictive validity (the ability to accurately predict outcomes or symptoms in humans; Sarter et al., 1992; Einat et al., 2003; Arime et al., 2011). The animal model should also combine genetic tractability, tools to visualize and manipulate neurons *in vivo*, and the ability to translate findings to patients based upon conserved neurobiology.

Zebrafish have already been established as a powerful model for developmental biology and neuroscience. Zebrafish develop rapidly outside of the mother making it easy to collect and manipulate embryos. By 6 days, larval fish swim continuously, search for food, and are able to escape from predators thus demonstrating a range of behaviors. Zebrafish are transparent until larval stages allowing the study and manipulation of neural circuits at the cellular level in the intact brain (Fetcho and Liu, 1998). Furthermore, a large number of identified mutant lines, genetic tools such as TALENs (transcription activator-like effector nucleases) and zinc-finger nucleases to knock-out genes; Amacher, 2008; Huang et al., 2011; Sander et al., 2011), genetic ablation (Curado et al., 2007), optogenetics (Nagel et al., 2003; Zhang et al., 2007), and techniques to monitor neural activity (including calcium indicators and electrophysiology; Higashijima et al., 2003) have already been established. Although the formation, position, and function of neurotransmitter signaling pathways sometimes differ between zebrafish and other vertebrates, comparative studies are beginning to precisely map these differences, allowing the transfer of information gained in zebrafish to other species (Tropepe and Sive, 2003). Furthermore, a battery of tests for behavioral analysis of both larval and adult zebrafish has already been developed (Fero et al., 2011; Norton and Bally-Cuif, 2010; Norton et al., 2011). Although tools to study neuroscience are already available in other genetically tractable vertebrates such as mouse, the ease of generating large numbers of zebrafish and their transparency make them ideal for high-throughput analyses and imaging studies. As a model for behavioral neuroscience, the zebrafish is particularly useful for optogenetic dissection of the behavior, time-lapse analysis of neurotransmitter pathway formation during development and screening for novel therapeutic treatments. For example, the compensatory changes to neurotransmitter signaling pathways that occur following genetic manipulation can be examined in zebrafish. Abrogation of a single gene will most likely lead to the modification of multiple neurotransmitters. These fluctuations could be examined in two ways. The levels of neurotransmitters in the brain could be measured directly by either high pressure liquid chromatography (HPLC) or an enzyme-linked immunosorbent assay (ELISA). Alternatively, the alterations to neural circuits in the brain could be uncovered using calcium indicators driven by neurotransmitter-specific promoters [such as otpb. A for dopamine (DA; Fujimoto et al., 2011) or pet1 for serotonin (5-HT; Lillesaar et al., 2009)]. Neural activity (detected as flashes of calcium signaling) could be measured in fish lacking a functional copy of an ADHD-linked gene. This information might provide clues about the signaling pathways and brain areas underlying a phenotype and so provide avenues for future research.

The power of zebrafish to study developmental biology suggests that it may make important contributions to the study of genes associated with psychiatric disorders. The zebrafish homologs of genes linked to psychiatric disorders can be identified, and their basic function during neural development (related to neural circuit formation or behavior for example) analyzed following manipulation. For example, a zebrafish line could be created which harbors a mutation mimicking the situation in human patients. This could include replacing the wild-type zebrafish transcript with a humanized form of the gene or knocking the gene down by TALEN or morpholino injection. The developmental and behavioral phenotype of the manipulated fish could then be characterized. Environmental interactions with these models could be studied by applying standardized environmental manipulations – for example, by stressing the fish before testing behavior (Amir-Zilberstein et al., 2012), growing embryos in a hypoxic environment (Marks et al., 2005) or treating with alcohol or nicotine (see below). The data generated by this approach could then be verified in rodents and if similar results are obtained then clinical trials would be initiated. Apparent behavioral similarities between animals (which are often used to develop models of human diseases) may not ensure that the same underlying process is being measured, since each behavior might not serve the same purpose in all species. In contrast to this, developmental models of psychiatric disorders may have improved construct validity if the model is based upon the same underlying changes to development which lead to expression of the disease.

In the following section, I will briefly summarize zebrafish models of ADHD, schizophrenia, ASDs, and X-linked mental retardation (XLMR).

## ZEBRAFISH MODELS OF ADHD

Attention-deficit/hyperactivity disorder is a common neuropsychiatric disorder that is characterized by developmentally inappropriate inattention, hyperactivity, and impulsivity. It affects around 3–5% of children worldwide regardless of nationality or cultural setting (Swanson et al., 1998; Polanczyk et al., 2007). The symptoms of ADHD persist into adulthood in about 50% of cases and can lead to a reduction in the quality of the sufferer’s life including impairment of academic, behavioral, and social performance (Barkley et al., 2006; Schmidt and Petermann, 2009). ADHD patients are also more likely to suffer from other psychiatric disorders, including depression, anxiety, and substance use disorder (Molina and Pelham, 2003; Lesch et al., 2008; Sharp et al., 2009). Data from drug treatments and genetic analyses have suggested that alterations in DA and noradrenaline (NA; and to a lesser extent 5-HT and glutamate) signaling most likely underlie the symptoms of ADHD. For example, methylphenidate (MPH), an amphetamine like compound that increases both DA and NA levels in the prefrontal cortex (Berridge et al., 2006) can be used to manage ADHD and so has orientated research toward monoaminergic signaling. ADHD patients are thought to have a reduction of dopaminergic signaling in the prefrontal cortex. Other brain areas which have been connected to ADHD include the striatum (caudate nucleus and putamen) the parietal cortex and both the vermis and the inferior lobes of the cerebellum (Berquin et al., 1998; Arnsten, 2007; Bush, 2010; Rubia, 2011).

Multiple DA pathway-related genes have been linked to ADHD. Association with polymorphisms in the gene encoding the DA D4 receptor (*DRD4*; Ebstein et al., 1996; LaHoste et al., 1996), the DA D5 receptor (*DRD5*; Hawi et al., 2002), and the DA transporter gene (*DAT*/*SLC6A3*; Cook et al., 1995) have been reported. Most studies of *DAT* have focused on a 40 base-pair variable number tandem repeat (VNTR) found in the 3^′^ untranslated region (UTR) of the gene (Vandenbergh et al., 1992; Curran et al., 2001; Purper-Ouakil et al., 2005). There is also some evidence associating the DA synthesis enzyme *Dopamine-beta hydroxylase* (*DBH*) and the disease (Comings et al., 1996). In the 5-HT pathway, the 5-HT synthesis enzymes *TPH1* and *TPH2* (Walitza et al., 2005; Li et al., 2007) and the 5-HT transporter gene (*SERT/SLC6A4*; Gizer et al., 2009) have all been linked to ADHD formation. Recent GWAS and candidate gene studies have also identified polymorphisms in genes that are involved in cell adhesion (including *ASTN2* and *CDH13*) and synaptogenesis (S*NAP25*, *CTNNA2*, and *KLRN*; Faraone et al., 2005; Lesch et al., 2011). Thus, as well as being caused by direct modification of neurotransmitter signaling, ADHD may be triggered by more general alterations in brain formation, including cell-signaling, morphogenesis, and migration during development.

There is currently only one study that has reported the use of fish to directly study an ADHD-linked gene (Lange et al., 2012). Lange et al. (2012) have studied *latrophilin3.1*, a zebrafish homolog of the human ADHD-susceptibility gene *LATROPHILIN3*. *LPHN3* was identified by linkage analysis of a genetically isolated European population in Columbia, followed by fine-mapping of several North American and European populations (Arcos-Burgos et al., 2010). Recent research has identified two families of endogenous ligands for Latrophilin3, the Teneurins and the FLRTs (Fibronectin Leucine-rich repeat transmembrane proteins; Silva et al., 2011; O’Sullivan et al., 2012). *latrophilin3.1* is one of two zebrafish homologs of human *LPHN3*, both of which are expressed in differentiated neurons throughout the brain up to 6 days post fertilization. *lphn3.1* morphants show an increase in the distance swum at 6 days, a hyperactive phenotype. This hyperactivity is also maintained during the night suggesting a permanent increase in locomotion compared to animals injected with a control morpholino. Furthermore, *lphn3.1* morphants also show an increase in the number of bursts of acceleration while swimming indicative of motor impulsivity. Both hyperactivity and motor impulsivity can be rescued by applying the ADHD treatment drugs MPH and atomoxetine. Acute treatment of either drug had no effect on control-injected larval behavior at the doses used (10 μM MPH or 1 μM atomoxetine for 1 h), but rescued morphant behavior bringing locomotion back to control levels. *lphn3.1* morphants also display a parallel reduction of dopaminergic cells in the posterior tuberculum (PT), a prominent group of dopaminergic neurons in the ventral diencephalon that controls larval locomotion (Bretaud et al., 2004; Sallinen et al., 2009; Tay et al., 2011). Recent analysis of the molecular signature and projection pattern of zebrafish PT neurons suggests they are similar to mammalian hypothalamic A11 DA neurons (Tay et al., 2011). Similar to A11, individual PT DA neurons project both anteriorly and posteriorly with the majority of projections (80%) going to the spinal cord (Ryu et al., 2007; Tay et al., 2011). Lesion of A11 DA neurons in rat causes a restless legs syndrome (RLS)-like hyperactivity phenotype and comorbidity between RLS and ADHD has been observed (Cortese et al., 2005). Thus common genes and neural circuits might underlie both diseases in mammals. Regardless of the homology of the zebrafish PT with other species, *lphn3.1* appears have a critical role in controlling the development of dopaminergic neurons (Lange et al., 2012), a finding which has recently been confirmed in mice (Wallis et al., 2012).

## ZEBRAFISH MODELS OF SCHIZOPHRENIA

Schizophrenia is a severe psychiatric disorder whose symptoms include mood changes (such as delusions and hallucinations), disorganization of thought, agitated body movements, anhedonia, depression, speech problems, and a lack of motivation. Although schizophrenia is thought to be caused by defects in early brain development (Weinberger, 1995), disease symptoms often do not appear until the second or third decade of life (typically between 16 and 30 years of age). Schizophrenia affects around 1% of the adult population in the USA according to the National Institute of Mental Health. Twin studies give a heritability estimate of about 81% for schizophrenia, and an environmental effect (including variables such as diet, parenting style, and exposure to toxins or teratogens) of around 11% (Sullivan et al., 2003). A large number of schizophrenia cases are sporadic, appearing for the first time in a family with no previous history of the disease (Xu et al., 2008). Rare *de novo* CNVs are one mechanism that can account for sporadic cases. In agreement with this, schizophrenia patients carry more CNVs than the general population and also have a higher than normal *de novo* mutation rate (Girard et al., 2011, 2012).

Neurobiological studies have identified defects in the frontal and temporal lobes of schizophrenia patients. Many schizophrenics also have enlarged cerebral ventricles linked to a 5–10% reduction in gray matter volume in the absence of gliosis, suggesting that loss of tissue is not caused by degeneration (Mueser and McGurk, 2004). Both dopaminergic and glutamatergic neurotransmission has been linked to the disease. For example, a reduction of blood flow in the prefrontal cortex related to DA activity has been documented in schizophrenia patients (Mueser and McGurk, 2004). Many genes have been connected to the susceptibility to suffer from schizophrenia. For example, genes with particularly strong linkage to the disease include *DISRUPTED IN SCHIZOPHRENIA1* (*DISC1*), *NEUREGULIN1*, *DYSTROBREVIN BINDING PROTEIN1* (*DTNBP1*), *KIF1*, *KIF17*, *SHANK3*, and *NOTCH4* (Tarabeux et al., 2010; Girard et al., 2012). Developmental processes which might be affected include genes which affect neural proliferation, differentiation, and migration during development or abnormal myelination in the schizophrenic brain (Flynn et al., 2003).

Although it is not possible to study the positive symptoms of schizophrenia such as disordered thought, delusions, and hallucinations in zebrafish, the basic developmental function of genes linked to this disease can still be studied. For example, schizophrenia candidate genes may be important for neurogenesis, neuronal migration, and cell fate determination (Morris, 2009). Burgess and Granato (2007) have developed a schizophrenia endophenotype in zebrafish: prepulse inhibition (PPI). PPI is a type of sensorimotor gaiting (a reduction of startle response that occurs when preceded by a weak non-startling stimulus) that is impaired in schizophrenic patients (Braff et al., 2007). Zebrafish PPI is modified by both apomorphine and ketamine [which affect DA and *N*-methyl-D-aspartate (NMDA) signaling respectively] and thus appears to be mediated by similar neurotransmitters as in other animals. Five novel mutant lines with abnormal PPI responses were also identified in the same study. Characterization of these mutants might give novel insights into the genes and brain areas that control behaviors linked to schizophrenia.

One of the most intensely studied schizophrenia-related genes is *DISC1*, which was first identified in a Scottish pedigree displaying high incidence of depression, schizophrenia, and bipolar disorder (Millar et al., 2000). *DISC1* has subsequently been shown to be involved in neurogenesis, neural migration, axon growth, synaptogenesis and function, dopaminergic neuron function, and cell–cell adhesion. Studies of *disc1* in zebrafish have provided novel information about the function of this gene. De Rienzo et al. (2011) have shown that *disc1* has a critical role in both the canonical (β-catenin-mediated) and non-canonical Wnt signaling pathways during embryonic development. Furthermore, different humanized forms of *disc1* have been shown to activate different signaling pathways in zebrafish. For example, Ala38Val, Arg264Gln, and Leu607Phe *disc1* variants interact with canonical Wnt signaling, whereas a Ser704Cys variant modifies neuronal migration via the cytoskeletal genes *nde1* and *dixdc1* (Singh et al., 2011). *disc1* has also been shown to control oligodendrocyte proliferation, a phenotype that can also be generated by inhibiting the schizophrenia-susceptibility genes *neuregulin1* and its receptor *erbb4* (Wood et al., 2009).

There have been several studies that have characterized schizophrenia-related genes in zebrafish. For example, the connection between Akt signaling, dopaminergic neurotransmission, and schizophrenia has been investigated. Cheng et al. (2013) have used zebrafish to study *rgs4*, a regulator of G-protein signaling family that is expressed in the developing nervous system. Loss of gene function causes both neurite outgrowth defects in the hindbrain and spinal cord and a reduction of motility. Activation of Akt signaling can rescue the outgrowth phenotype in the spinal cord but not hindbrain, indicating that Akt signaling is required for some *rgs4*-mediated axon formation (Cheng et al., 2013). Souza et al. (2011) have further examined the connection between Akt signaling and dopaminergic neurotransmission in the developing brain. Since alterations in Akt signaling have also been linked to schizophrenia, this strengthens the suggestion that altered dopaminergic signaling during development might underlie the disease. Other human disease-causing genes have been studied as well. Knock-down of the synaptically expressed schizophrenia-susceptibility gene *kinesin17* (*kif17*) causes a severe phenotype in zebrafish embryos including stunted development and a curly tail phenotype suggesting that *kif17* is active during embryonic development (Tarabeux et al., 2010). Finally, treatment of zebrafish with the NMDA receptor antagonist MK-801 causes several behavioral alterations including changes to social interaction, hyperactivity, and amnesia, which have been interpreted as being schizophreniform (schizophrenia-like; Chen et al., 2010; Seibt et al., 2010, 2011; Echevarria et al., 2011) and are similar to the behavioral changes elicited by these psychotics in rodents.

In summary, although it is not possible to model some of the more complex psychological symptoms of schizophrenia in fish, several recent studies have given insights into some of the basic processes underlying this disease.

## ZEBRAFISH MODELS OF AUTISM SPECTRUM DISORDER

Autism spectrum disorder encompasses a range of psychiatric diseases including autism (the severest form of ASD), Rett’s syndrome, and Asperger’s syndrome. The symptoms of ASD are highly variable between patients making it difficult uncover the genetic and neurobiological changes that underlie these disorders. Broadly speaking, the main symptoms of ASD include alterations in social behavior, repetitive behavior, and language development that appear before 3 years of age (Lord et al., 2000). There is a heritability estimate of between 70 and 80% for ASD (Bailey et al., 2005; Rosenberg et al., 2009). However, the number of people thought to suffer from ASD is increasing over time which might be due to a shift in the diagnostic criteria used (Lord, 2010). Although it is not possible to study complex behaviors such as language development in fish, a few recent studies have provided information about the function of ASD-linked genes during neural development.

Both common and rare (*de novo*) genetic variants, acting in combination with environmental influences, are associated with ASD. ASD-linked polymorphisms are predominantly found in genes that regulate synapse development, cell proliferation, neural migration, and neural projection. Rare mutations seem to account for around 10% of cases of ASD (Geschwind, 2008). It has been suggested that there may be a different genetic basis for simplex families (where only one child is affected and rare mutations are common) and multiplex families (where multiple genetic variants lead to ASD expression in several family members; Geschwind, 2008). For example, there is a higher frequency of CNVs in ASD families with only one affected child compared to those with two or more ASD children (Toro et al., 2010). Interestingly, there is also some data suggesting that rare and common variants interact to trigger ASD symptoms (Ben-David and Shifman, 2012). Genes which have been linked to ASD include the Fragile X gene *FRAGILE X MENTAL RETARDATION 1* (*FMR1*), the GABA_A_ β 3 subunit gene *GABRB3* as well as SHANK3, *TSC1*, *NEUROLIGIN3* and *NEUROLIGIN4*, *PTEN* and *CNTNAP2* (Cook and Scherer, 2008; Geschwind, 2008, 2011; Lord, 2010) amongst others. The neurobiology of ASD is not well understood, but most likely includes brain areas which are needed for the control of social behavior and language. For example, altered connections between the frontal lobe (the orbitofrontal cortex) and temporal lobe (including the superior temporal gyrus and temporal polar cortex) may mediate some symptoms of the disease (Geschwind, 2011). Other important brain areas include the cerebellum, brainstem, and limbic areas including the hippocampus, amygdala, septal nuclei, and the anterior cingulate cortex (Lord et al., 2000). Finally, autism has also been related to megalencephaly, an overall increase in brain size in some patients.

Since the symptoms of ASD first occur during neural development it is possible to study the early function of ASD-related genes in this species (Tropepe and Sive, 2003). There are only a few research articles reporting the use of zebrafish in this type of research. In humans, a CNV at 16p11.2 has been associated with susceptibility to suffer from several disorders, including ASD, epilepsy, autism, and schizophrenia. The 16p11.2 CNV has been studied in zebrafish by two groups (Blaker-Lee et al., 2012; Golzio et al., 2012). In a landmark study, Golzio et al. (2012) analyzed 24 of the genes contained within this CNV and identified one, *KCDT13*, as most likely being causative for the disease. Overexpression of *kcdt13* (by mRNA injection) led to microcephaly, whereas gene-specific morpholino knock-down caused an increase in head size, likely due to alteration of the cell cycle in both cases. This study provides a neat demonstration of the use of zebrafish to identify disease-causing variants within large genomic regions. Gauthier et al. (2010) have used morpholinos to knock-down *SHANK3*, a gene linked to schizophrenia and ASD in human patients. *shank3* morphant zebrafish show decreased swimming after being touched, a phenotype which can be rescued with a wild-type (but not a mutant) version of the corresponding rat gene. Therefore, this study validated alterations to *shank3* as being important in the control of behavior, with potential implications for both schizophrenia and ASD.

## ZEBRAFISH MODELS OF MENTAL RETARDATION

Mental retardation constitutes a significant impairment of cognitive function that can be symptomatic of a number of underlying neural defects. In this review, I have chosen to focus on XLMR (also called Fragile X syndrome, FXS) a common inherited form of mental retardation which affects around 1 in 4000 people. However, zebrafish have also been used to examine other types of mental retardation (for recent studies, refer to Komoike et al., 2010; Song et al., 2010; Brockschmidt et al., 2011; Friedrich et al., 2012; Veleri et al., 2012; Aspatwar et al., 2013). The symptoms of FXS include mental retardation, epilepsy, autistic-like behavior, attention deficits, macroorchidism, and mild craniofacial defects which have been linked to the maturation of dendritic spines during development.

Similar to schizophrenia and ASD, it is likely to be impossible to model the cognitive symptoms of mental retardation in fish. However, several zebrafish groups have analyzed XLMR-linked genes and provided information about their role during development. For example, XLMR is most often caused by mutations in the *FMR1* gene, which contains a variable CGG trinucleotide repeat sequence in its 5^′^ UTR (Verkerk et al., 1991). Once this repeat passes 200 copies, the gene becomes hypermethylated and loss of function occurs. Interestingly, this methylation appears to be mediated by a microRNA which is present in the 3^′^ UTR of *FMR1*, an observation which has been experimentally confirmed in zebrafish (Lin et al., 2006). Injection of a construct targeting the anti-fmr1 miRNA causes abnormal neural morphology and an increase in the size of the head (Lin et al., 2006).

Studies of the zebrafish homolog of the *FMR1* gene have given equivocal results. Intriguingly, different phenotypes were observed when comparing morpholino-mediated knock-down and a stable *fmr1* mutant line. Morphant larvae show craniofacial defects, hydrocephaly, and pericardial edema as well as abnormal branching of the trigeminal ganglion and lateral longitudinal fasciculus (Tucker et al., 2006). However, in contrast to this, *fmr1* mutant larvae are adult viable and show no detectable morphological differences (Den Broeder et al., 2009). Thus, it is clear that further studies are needed in order to fully understand the function of this gene during development. Qi et al. (2010) studied the XLMR gene *phf8* during zebrafish development. *phf8* is a mono-methyl histone H4 lysine 20 demethylase that is expressed in the developing head and jaw of zebrafish at 1 day. Loss of gene function causes severe disruption to development of these structures, most likely caused by increases in apoptosis (Qi et al., 2010). Histone methylation has also been linked to XLMR in a study of the *smcx/jarid1c* gene, a H3K4 demethylase (Iwase et al., 2007). Loss of *smcx* function during development leads to increased cell death and a smaller brain as well as a reduced number of dendrites. Together, both of these studies suggest that epigenetic events might play an important role in the etiology of XLMR. Another XLMR-linked gene, *rbmx*, shows strong expression in the anterior developing embryo including the brain (Tsend-Ayush et al., 2005). Knock-down of *rmbx* leads to a reduction in body size, pericardial edema, hydrocephaly, and reduced motility (Tsend-Ayush et al., 2005). Finally, the non-syndromic mental retardation genes *il1rapl1a*, *il1rapl1b*, and *il1rapl2* (zebrafish homologs of human *IL1RAPL*, which is located on the X chromosome) are widely expressed throughout the brain. Knock-down of one of the homologs, *ilrapl1b*, inhibits presynaptic differentiation during development (Yoshida and Mishina, 2008).

There are several learning and choice discrimination tests that have been developed for adult zebrafish which could be used to test possible cognitive functions of mental retardation-associated genes. For example, Brennan and colleagues have developed a 3-choice serial reaction time task (3CSRTT) that can be used to measure impulsivity (Parker et al., 2012). The 3CSRTT is measured in a tank that has a green light-emitting diode (LED) on one side and three yellow LEDs in separate compartments on the other. Following illumination of the green LED, adult zebrafish are taught to only enter the compartment where the yellow LED is switched on. The correct execution of this behavior is reinforced with a food reward. Following a training period in which the fish learns to associate the yellow light with a reward the 3CSRTT can begin. The green stimulus LED is first activated and is then followed by a 10-s intertrial interval (ITI). Following this pause, one of the yellow LEDs is lit and the fish is rewarded with food upon entering the correct compartment. However, entry into any compartment before the end of the ITI, perhaps indicative of impulsivity, will result in a punishment (a 10-s time-out with no food). Entry into an incorrect compartment on the other side (i.e., one in which the yellow LED is not illuminated) will also trigger the punishment. Other tests of learning and memory, including associative-, avoidance-, and spatial memory have also been established (reviewed in Norton and Bally-Cuif, 2010). Although these tests appear to be promising in to study zebrafish cognition, they have mostly only been tested on wild-type fish and so need further validation (for example, in fish lacking the function of a disease-linked gene) before they can be proposed as retardation-linked phenotypes.

## FUTURE DIRECTIONS FOR RESEARCH INTO PSYCHIATRIC DISORDERS USING ZEBRAFISH

The functions of genes linked to psychiatric disorders are still relatively understudied in zebrafish. However, the power of zebrafish as a model organism to study neural development and behavior suggest that there are several areas in which the zebrafish has the potential to provide more information related to these diseases, as discussed below.

## ENVIRONMENTAL CONTRIBUTIONS TO DISEASE ONSET

Since zebrafish embryos develop outside of their mother, it is easy to manipulate their environment and so carry out G × E studies. Although it is not possible to model adverse psychosocial environments (such as parenting styles) in fish, other environmental factors including exposure to alcohol or nicotine could easily be studied. Ethanol exposure during development causes a range of phenotypes depending on the timing and concentration used, making it difficult to get an overview of the mechanism underlying the action of this drug, or how it might relate to alcohol-related changes to behavior in humans. At concentrations greater than 2%, ethanol causes gross body abnormalities including cardiac edema, cyclopia, and dysmorphia (Blader and Strahle, 1998; Sylvain et al., 2010). Lower concentrations trigger behavioral alterations in the absence of obvious morphological changes. For example, transient exposure to ethanol can lead to either locomotor hyperactivity (Tal et al., 2012), a reduction of swimming coupled to defects in secondary motorneuron development and synaptogenesis (Sylvain et al., 2010, 2011) or reduced shoaling in adult fish (Fernandes and Gerlai, 2009; Buske and Gerlai, 2011). Embryos which are treated with nicotine during development tend to be smaller, with a permanent delay and disruption of secondary motorneuron development (Svoboda et al., 2002; Menelaou and Svoboda, 2009). Nicotine also causes a transient increase in swimming speed, followed by a long-lasting reduction in both startle response and swimming speed, a phenotype which is most likely mediated via activation of nAChRs (Svoboda et al., 2002; Eddins et al., 2010).

One of the difficulties of designing G × E studies, highlighted by the examples related to ethanol and nicotine, will be to mimic the situation in nature as closely as possible. Although environmental alterations may modify the severity of an obvious pre-existing phenotype, in some cases environmental influences would be expected to trigger disease-related symptoms in a genotype that initially looks normal. One way to address this issue would be to develop a zebrafish transgenic line that contains a humanized form of the disease-risk gene and then subject embryos to different environmental conditions (such as varying concentrations of ethanol or nicotine exposure) before measuring behavior. Although such an approach is not more technically difficult than starting with a measurable behavioral change, it would require researchers to have more confidence when developing the assay.

## SCREENING FOR NOVEL DRUGS

Zebrafish are a good model system for pharmacological studies, since compounds can be diluted in the embryo medium, and larvae are transparent meaning that internal organs can be visualized throughout development (Peterson et al., 2000). Furthermore, larvae are small, easy to generate in large numbers and easy to manipulate making them ideal for high-throughput work. However, since screens that use behavior as a read-out are difficult to design and implement efficiently (particularly if focusing on complex behaviors other than locomotion), it might be easier to carry out an initial pre-screen of a disease endophenotype. For example, zebrafish lacking the function of the ADHD-linked gene *lphn3.1* show a reduction and displacement of dopaminergic neurons in the PT of the diencephalon (Lange et al., 2012). Pre-screening libraries for compounds which can rescue this phenotype might speed up the screening process, with a subset of promising drugs then being re-tested to assess their behavioral function.

Two studies have combined analysis of underlying chemical structure and high-throughput screening of chemical libraries to look for alterations to the photomotor response, an embryonic motor response to a series of light flashes (Kokel et al., 2010; Laggner et al., 2012). This assay can be performed in a 96-well plate, and can be scaled up to analyze around 5000 novel chemicals every day. Rihel et al. (2010) uncovered the effect of drugs on locomotor activity across different sleep/wake cycles, providing novel information about neuroactive drugs which can affect behavior. In this study, chemicals were categorized according to their “behavioral fingerprint,” a combination of their chemical structure and their ability to modify aspects of behavior, thus permitting prediction of the function of novel chemicals.

A screen for novel anti-epileptic drugs was conducted in 2-day-old zebrafish embryos by Baxendale et al. (2012). Although not a developmental psychiatric disorder, this work further demonstrates the utility of zebrafish for drug identification. Exposure of embryos to the convulsant pentylenetetrazole (PTZ) upregulates expression of the immediate-early gene *fos* in the brain. The level of *fos* expression (assayed by *in situ* hybridization) was then used to screen a bioactive small-molecule library with a hit rate of 2.3% for anti-convulsive drugs (Baxendale et al., 2012). Using a similar approach, Baraban (2007) screened for novel zebrafish mutant lines which are resistant to the seizure-inducing properties of PTZ at 7 days post fertilization. Six novel mutant lines were identified, raising the possibility of uncovering novel genes with a seizure-protective function. The effects of the stimulants ethanol, amphetamine, and cocaine on larval swimming behavior have also been reported (Irons et al., 2010). Whilst not high-throughput, this study demonstrates the ease of screening motor behavior in 96-well plates, meaning that this paradigm could potentially be used to look at hyperactivity or startle, endophenotypes related to psychiatric disorders.

Although zebrafish constitute a power model system for drug screens, further experiments will be required before data can be translated to human patients. In a first step, the results will need to be verified in a second animal model – most likely a knock-out mouse harboring a similar genetic lesion. Analysis of further disease models with and without drug treatment will provide information about selectivity of the chemical compound for one or more signaling pathways. A drug with a similar profile in both fish and mouse will represent a very promising target for clinical trials as long as no toxic effects are detected.

## CONCLUSION

A full understanding of the basis of psychiatric disorders requires the characterization of complex behaviors such as thought, feeling, and emotion, most likely through translational experiments in a combination of model organisms such as zebrafish and rodents. However, the suitability of zebrafish for developmental studies, and the genetic tools which are available to manipulate them suggest that the early development function of psychiatric disorder-linked genes can be studied in this model organism. Studies of genes linked to ADHD, schizophrenia, ASD, and mental retardation that have been performed in zebrafish have already demonstrated the validity of this approach. I look forward to future developments in this exciting field.

## Conflict of Interest Statement

The author declares that the research was conducted in the absence of any commercial or financial relationships that could be construed as a potential conflict of interest.
